# Characterization of Human Mesenchymal Stem Cells from Different Tissues and Their Membrane Encasement for Prospective Transplantation Therapies

**DOI:** 10.1155/2019/6376271

**Published:** 2019-03-03

**Authors:** Eva Schmelzer, Daniel T. McKeel, Jörg C. Gerlach

**Affiliations:** ^1^Department of Surgery, School of Medicine, University of Pittsburgh, McGowan Institute for Regenerative Medicine, Pittsburgh, PA 15203, USA; ^2^Department of Bioengineering, School of Medicine, University of Pittsburgh, McGowan Institute for Regenerative Medicine, Pittsburgh, PA 15203, USA

## Abstract

Human mesenchymal stem cells can be isolated from various organs and are in studies on therapeutic cell transplantation. Positive clinical outcomes of transplantations have been attributed to both the secretion of cytokines and growth factors as well as the fusion of donor cells with that of the host. We compared human mesenchymal stem cells from six different tissues for their transplantation-relevant potential. Furthermore, for prospective allogenic transplantation we developed a semipermeable hollow-fiber membrane enclosure, which would prevent cell fusion, would provide an immune barrier, and would allow for easy removal of donor cells from patients after recovery. We investigated human mesenchymal stem cells from adipose tissue, amniotic tissue, bone marrow, chorionic tissue, liver, and umbilical cord. We compared their multilineage differentiation potential, secretion of growth factors, and the expression of genes and surface markers. We found that although the expression of typical mesenchymal stem cell-associated gene THY1 and surface markers CD90 and CD73 were mostly similar between mesenchymal stem cells from different donor sites, their expression of lineage-specific genes, secretion of growth factors, multilineage differentiation potential, and other surface markers were considerably different. The encasement of mesenchymal stem cells in fibers affected the various mesenchymal stem cells differently depending on their donor site. Conclusively, mesenchymal stem cells isolated from different tissues were not equal, which should be taken into consideration when deciding for optimal sourcing for therapeutic transplantation. The encasement of mesenchymal stem cells into semipermeable membranes could provide a physical immune barrier, preventing cell fusion.

## 1. Introduction

Mesenchymal stem cells (MSCs) have been isolated from various fetal and adult organs. Friedenstein et al. [1–3] first described mouse bone marrow MSCs and their multilineage differentiation potential. The multilineage differentiation potential of adult human MSCs from bone marrow was described by Pittenger et al. [4]. Since then, human MSCs from various organs have been described and transplanted clinically in multiple fields of applications [5, 6]. For autologous transplantation, the foremost important practical aspect is for certain the ease of sourcing. This makes adipose, skin, or bone marrow a more obvious choice than, for example, liver, placenta, or umbilical cord. Adipose tissue-derived MSCs can be obtained by liposuction under general anesthesia, while an iliac crest bone marrow sample can be obtained in a physician's office under local anesthetic. This makes obtaining bone marrow MSCs much less invasive than adipose-derived MSCs. Another important aspect for clinical applications is the potentially different capability of MSCs from different tissues to support the local environment by the release of growth factor and cytokines. More recently, the release of exosomes and microvesicles from MSCs has been investigated for cell-free therapies (for review, see [7]).

A variety of different growth factors, cytokines, exosomes, and microvesicles have been found to be secreted or taken up by human MSCs; sphingolipids and their receptors were shown to contribute to MSCs functioning and to regulate the course of transplantation (for reviews, see [8–10]). For example, Schinköthe et al. [11] analyzed 120 cytokines and growth factors in the cell culture medium of human bone marrow-derived MSCs. From these, 44 were found to be secreted into the medium and 40 were taken up from the medium. Shen et al. [12] analyzed 16 growth factors and cytokines that were secreted by human umbilical cord-derived MSCs.

Effects of transplantation of MSCs have not been only related to the release of growth factors and cytokines, but also to mitochondrial transfer [13] as well as fusion [14, 15] of donor MSCs with host cells. For allogenic MSC transplantation, the option to remove donor MSCs after patient recovery is of interest in order to avoid long-term effects due to potential cell fusion and immunological complications. The encasement of MSCs should occur in porous structures with adequate pore sizes as to allow the unrestricted release of secreted molecules but to prevent cell release. Different methods for encapsulation of cells for transplantation were described; cells have been commonly encapsulated in alginate [16–20] or other types of gel-like embedding matrices [21–23], but unusual approaches included, for example, the use of silk [24]. We developed a semipermeable membrane hollow-fiber assembly that provides adequate and adjustable pore sizes, allows for easy filling, can be variable in length to accommodate various amounts of cells, allows for potential removal of cells if necessary, and is made of biomedical grade materials that have been applied* in vivo *and* in vitro.*

In our study, we investigated two different aspects relevant for potential clinical transplantation. These included a comparison of MSCs derived from different donor sites and the* in vitro* assessment of a fiber encasement for prospective clinical implantation. We investigated the expression of typical positive and negative surface markers that had been defined as minimum criteria [25]. We compared their chondrogenic, osteogenic, and adipogenic differentiation potential. Moreover, we examined if the encasement in fibers affected lineage-specific gene expression, cell viability, and secretion of growth factors.

## 2. Materials and Methods

### 2.1. Cell Culture

Human mesenchymal stem cells from adipose (AD), amniotic (AM), bone marrow (BM), chorionic (CH), liver (LI), and umbilical cord matrix (UC) tissues were obtained frozen (Sciencell, Carlsbad, CA; ATCC, Manassas, VA; Promocell, Heidelberg, Germany). Cells were seeded at a density of 5,000 cells per cm^2^ and cultured for two to three passages in T-flasks with human StemMACS MSC Expansion Media XF (Miltenyi Biotec, Bergisch Gladbach, Germany) (which was serum- and xenobiotic-free), including manufacturer-supplied supplement, 100 units/mL penicillin, 100 *µ*g/mL streptomycin, and 0.25 *µ*g/mL fungizone (Gibco/Thermo Fisher Scientific, Pittsburgh, PA). Cell viabilities and numbers were monitored by Trypan Blue exclusion in a Neubauer Chamber.

### 2.2. Flow Cytometric Analyses

Cells propagated in culture for two or three passages were analyzed for their expression of typical mesenchymal and lineage-specific surface markers. Single cell suspensions were stained in Brilliant Stain Buffer (Becton Dickinson, Franklin Lakes, NJ) containing 10% human FcR block (Miltenyi Biotec). Controls included nonstained cells and cells incubated with corresponding isotype controls. Antibodies and isotypes (all Becton Dickinson) were as follows: APC mouse IgG1 anti-human CD73, mouse IgG1-APC, FITC mouse IgG1 anti-human CD105, mouse IgG1-FITC, BV421 mouse anti-human CD90, mouse IgG1-BV421, PE mouse anti-human CD34, mouse IgG1-PE, BUV395 mouse IgG1 anti-human CD45, and mouse IgG1-BUV395. Cells were analyzed in a FACS Aria II flow cytometer (Becton Dickinson). Compensation beads (Becton Dickinson) were used to compensate potential spectral fluorochrome overlap. Cell debris and cell doublets were excluded by applying an initial forward versus side scatter gate. Raw data were analyzed using FlowJo software version 10.4.1 (FlowJo, Ashland, OR).

### 2.3. Multilineage Differentiation* In Vitro*

Cells were induced in culture to adipogenic, chondrogenic, and osteogenic differentiation. All differentiation cultures included control cultures that received regular MSC expansion medium.

For adipogenic differentiation, cells were cultured for 21 days in Mesencult Adipogenic Differentiation Medium (Stem Cell Technologies, Vancouver, Canada) including 100 units/mL penicillin, 100 *µ*g/mL streptomycin, and 0.25 *µ*g/mL fungizone (Gibco/Thermo Fisher Scientific). Cells were fixed with 4% PFA. Lipids were detected with Oil Red O Stain (Sigma-Aldrich, St.-Louis, MO), and cells were counterstained with hematoxylin QS (Vector Laboratories, Burlingame, CA). Images were acquired with a phase contrast light microscope (InvertoskopC), equipped with a camera (AxioCam MRc) and software (Axiovision Vs40, V4.2.0.0) (Zeiss, Thornwood, NY).

For chondrogenic differentiation, 5x10^5^ cells were pelleted at 250 g for 5 min and cultured for 24 days in StemMACS ChondroDiff Medium (Miltenyi Biotec) including 100 units/mL penicillin, 100 *µ*g/mL streptomycin, and 0.25 *µ*g/mL fungizone (Gibco). Pellets were fixed with 4% PFA, embedded in optimum cutting temperature (OCT) compound (Sakura Finetek, Torrance, CA) and snap frozen in liquid nitrogen. Five to ten *µ*m frozen sections were stained for aggrecan. Sections were incubated with a primary anti-human mouse IgG1 aggrecan antibody (Santa Cruz Biotechnology, Dallas, TX), and a secondary AlexaFluor555 anti-mouse IgG1 goat antibody (Invitrogen/Thermo Fisher Scientific). Human cartilage sections (Amsbio, Cambridge, MA) were used as positive control. Cell nuclei were stained with 4',6-diamidino-2-phenylindole. Images were acquired with a Nikon Eclipse TE300 fluorescence light microscope (Tokyo, Japan) equipped with a ProgRes MF camera and software (Jenoptik, Jena, Germany).

For osteogenic differentiation, cells were cultured for ten days in StemMACS OsteoDiff Medium (Miltenyi Biotec) including 100 units/mL penicillin, 100 *µ*g/mL streptomycin, and 0.25 *µ*g/mL fungizone (Gibco). Cultures were fixed with methanol at -20°C for 5 min, and alkaline phosphatase activity was detected by incubation with SIGMA FAST BCIP/NBT substrate (Sigma-Aldrich) for 10 min. Images were acquired with a phase contrast light microscope (InvertoskopC), equipped with a camera (AxioCam MRc) and software (Axiovision Vs40, V4.2.0.0) (Zeiss, Thornwood, NY).

All images were assembled in Adobe Photoshop CS5 Extended Version 12.0 software (Adobe Systems, San Jose, CA).

### 2.4. Capillary Fiber Preparation and Cell Culture

**Figure 1:**
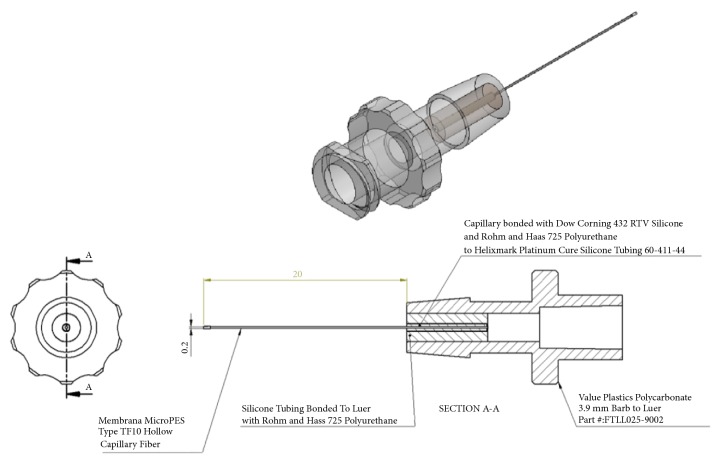
*Schematic of fiber construction.* Details of the fiber construction used for encased culture of mesenchymal stem cells, with dimensions given in mm.

Hydrophilic MicroPES Type TF10 hollow capillary fibers (Membrana, Wuppertal, Germany) with an inner diameter of 300 *µ*m were cut to a length of 20 mm. Membranes consist of biocompatible polyethersulfone and polyvinylpyrrolidone. The fibers have a molecular weight cut off of MW 400,000 (Daltons), permitting large proteins to also pass through the fibers. To enable easy filling of fibers with cells, we bonded fibers to Luer-Lock connectors bridged by silicone tubing (Figure 1). Fibers were bonded with 432 RTV silicone (Dow Corning, Midland, MI) and 725 Polyurethane (Rohm and Haas, Philadelphia, PA) to Helixmark Platinum Cure Silicone Tubing 60-411-44 (Freudenberg Medical, Kaiserslautern, Germany). The silicone tubing was bonded to a 3.9 mm polycarbonate Barb to Luer-Lock (Value Plastics, Fort Collins, CO). Open ends of fibers were sealed with 725 Polyurethane. Constructs were sterilized with ethylene oxide. Each fiber was filled with 50,000 mesenchymal cells and placed in a well of a 6-well tissue-culture plate containing 2.5 mL StemMACS MSC Expansion Media XF, including 100 units/mL penicillin, 100 *µ*g/mL streptomycin, and 0.25 *µ*g/mL fungizone. Controls of 50,000 cells were cultured directly in a well of a 6-well tissue-culture plate. Cells and culture media were harvested after seven days of culture. Cells in wells and fibers were directly lysed with RLT-buffer (Qiagen, Valencia, CA) containing 1% beta-mercaptoethanol (Sigma-Aldrich) for subsequent extraction of nucleic acids and gene expression analyses. Cell culture media samples were centrifuged to remove any potential cells or debris, and supernatants were stored at -20C for further analyses of protein secretion and lactate dehydrogenase (LDH) activity.

### 2.5. Gene Expression Analyses

Cells in control and fiber culture were lysed directly with RLT-buffer containing 1% beta-mercaptoethanol, and nucleic acids were isolated using shredder- and isolation-columns (AllPrep DNA/RNA-mini kit, Qiagen). RNA columns received DNA digestion by DNase treatment on columns. DNA columns were used to extract DNA for cell number correlations. Concentrations of nucleic acids were determined using Quant-iT Assay kits and Qubit fluorometer (Invitrogen/Thermo Fisher Scientific, Pittsburgh, PA). RNA was reverse transcribed to cDNA with the High-Capacity cDNA Reverse Transcription Kit (Applied Biosystems, Carlsbad, CA). Gene expression was analyzed with real-time PCR using the StepOnePlus system and software version 2.0, and predesigned TaqMan probe and primer assay mixes with gene expression master mix (Applied Biosystems). Beta-actin served as housekeeping gene for endogenous normalization. TaqMan assay mixes were for aggrecan (ACAN), adiponectin (ADIPOQ), beta-actin (ACTB), CD34, DMP1, CD45 (PTPRC), and CD90 (THY1).

Gene expression was quantified using the ddCt method. Positive controls included cDNA that had been reverse transcribed from RNA of human adult tissues of trachea, bone marrow, and fat (Biochain, Newark, CA). No template (water) was used as negative control. Each of the three biological samples was analyzed with two technical repeats.

### 2.6. Cell Viability

Viability of cells in conventional and fiber culture was determined by detecting the release of LDH enzyme in cell culture media samples. LDH is only released by damaged cells, so an increased enzyme activity correlates negatively with cell viability. Enzyme activity was measured using the QuantiChrom LDH Kit (BioAssay Systems, Hayward, CA) according to the manufacturer's instruction. The optical density (OD) at 565 nm of samples was read immediately and after 25 min, using a Synergy H1 Hybrid multimode microplate reader and Gen5 data analysis software (BioTek, Winooski, VT). LDH activity was calculated as IU/L. Water was used as negative control, day 0 culture medium was used as blank. Each of the three biological samples was analyzed with two technical repeats.

### 2.7. Enzyme-Linked Immunosorbent Assays

Concentrations of growth factors angiopoietin-2 (ANGPT2), basic fibroblast growth factor (bFGF), hepatocyte growth factor (HGF), and tissue inhibitor of metalloproteinase 2 (TIMP-2) were determined in cell culture media samples of conventional and fiber cultures using enzyme-linked immunosorbent assays (ELISAs) (RayBiotech, Norcross, GA). The OD at 450 nm of samples was read using a Synergy H1 Hybrid multimode microplate reader and Gen5 data analysis software (BioTek, Winooski, VT). Day 0 culture medium was used as blank. Each of the three biological samples was analyzed with two technical repeats.

### 2.8. Statistics

Data are given as means from n biological repeats ± standard deviation. Student's t-test was used to analyze statistically significant differences using Microsoft Excel version 16.14.1 (Redmond, WA).* P* values equal to or less than 0.05 were considered statistically significant.

## 3. Results

### 3.1. Expression of Surface Markers

**Figure 2:**
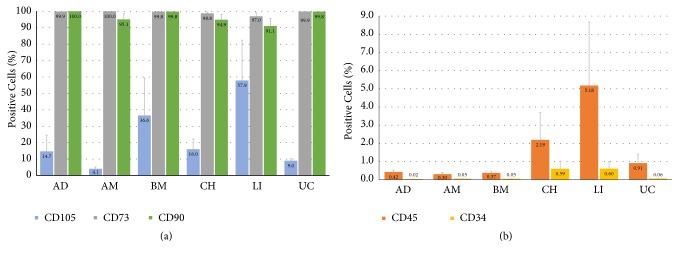
*Flow cytometric analyses of surface marker expression. *Mesenchymal progenitors from different organs were cultured for two or three passages and compared for their expression of typical (a) mesenchymal (CD105, CD73, and CD90) and (b) lineage-specific (CD45 and CD34) surface markers. AD: adipose tissue-derived; AM: amniotic tissue-derived; BM: bone marrow-derived; CH: chorionic tissue-derived; LI: liver-derived; UC: umbilical cord-derived.

We investigated the expression of surface markers that have been described as typical mesenchymal (CD105, CD73, and CD90) and lineage-specific (CD45 and CD34) in flow cytometry (Figure 2) of mesenchymal progenitors from different organs. We analyzed cells in conventional culture at passage two or three. We could confirm that MSCs from all organs were positive (> 90%) for CD90 and CD73 (Figure 2(a)). However, CD105 expression was low, and highly variable between MSCs from different organs; CD105 expression ranged from an average of 4.1% on AM MSCs to an average of 57.9% on LI MSCs. The expression of lineage-specific surface molecules (Figure 2(b)) was expected to be low. This was indeed the case for CD34 expression, which could be detected only in about 0.6% of LI and CH MSCs and to a much lesser percentage (less than 0.06%) in other MSCs. CD45 expression was low but could be detected in about 5% of LI and 2% of CH MSCs; in MSCs from other organs, the percentages of cells positive for CD45 were less than 1%.

### 3.2. Attachment, Morphology, and Proliferation Potential

**Figure 3:**
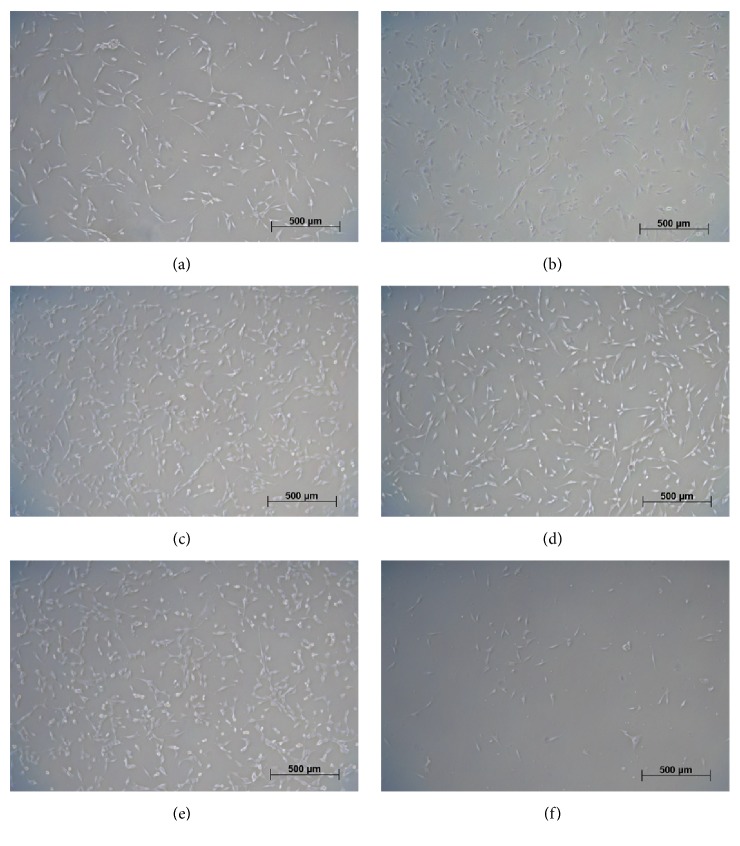
*Human mesenchymal stem cells in culture one day after plating.* (a) Adipose tissue-derived; (b) amniotic tissue-derived; (c) bone marrow-derived; (d) chorionic tissue-derived; (e) Liver-derived; (f) umbilical cord-derived. Phase contrast microscopy.

In general, one day after plating, the cryopreserved MSCs from all donor sites attached and exhibited a typical spindle-shaped morphology (Figure 3) and attachment efficiency, which had been defined as a typical minimum criterion for MSCs [25]. Attachment efficiency of UC MSCs appeared to be somewhat lower than that of other MSCs. We also compared the proliferation potential of all MSCs; population doubling times were calculated based on cell numbers during culture from initial plating through passage three. Population doubling times ranged on average from 51h to 78h, but no statistically significant difference could be observed between the different MSCs (AD: 51±32h; AM: 64±34h; BM: 52±39h; CH: 78±30h; LI:71±29h; and UC: 76±40h).

### 3.3. Multilineage Differentiation Potential

**Figure 4:**
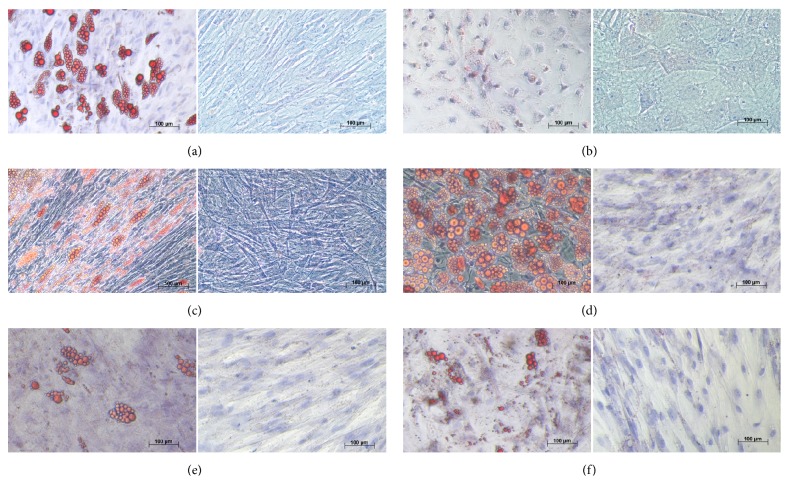
*Adipogenic differentiation of mesenchymal stem cells.* Human mesenchymal stem cells were subjected to adipogenic differentiation by using Mesencult Adipogenic Differentiation Medium for 21 days in culture. Lipids in cells were stained with Oil Red O Stain. (a) Adipose tissue-derived; (b) amniotic tissue-derived; (c) bone marrow-derived; (d) chorionic tissue-derived; (e) liver-derived; (f) umbilical cord-derived. Left panels: positive adipogenic-induced; right panels: negative controls noninduced. Phase contrast microscopy.

**Figure 5:**
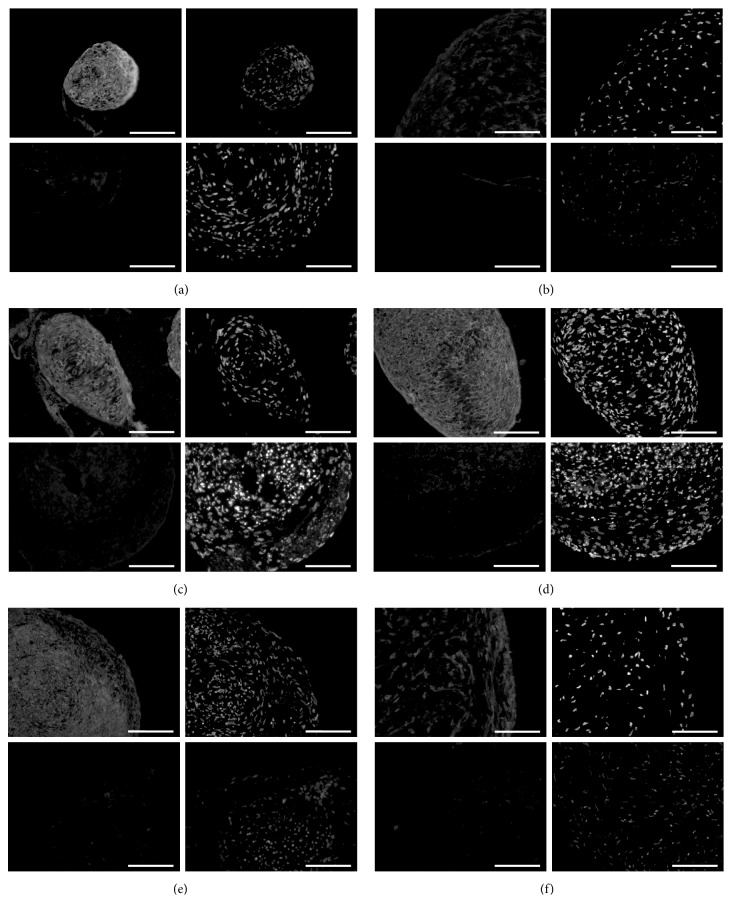
*Chondrogenic differentiation of mesenchymal stem cells.* Human mesenchymal stem cells were subjected to chondrogenic differentiation for 24 days in culture. (a) Adipose tissue-derived; (b) amniotic tissue-derived; (c) bone marrow-derived; (d) chorionic tissue-derived; (e) liver-derived; (f) umbilical cord-derived. For each assembly (a-f), upper panels: positive chondrogenic-induced spheres; lower panels: negative noninduced controls; left: chondrocyte-specific aggrecan stain; right: DAPI stain for cell nuclei. Fluorescence microscopy. Size bar: 150 *μ*m.

**Figure 6:**
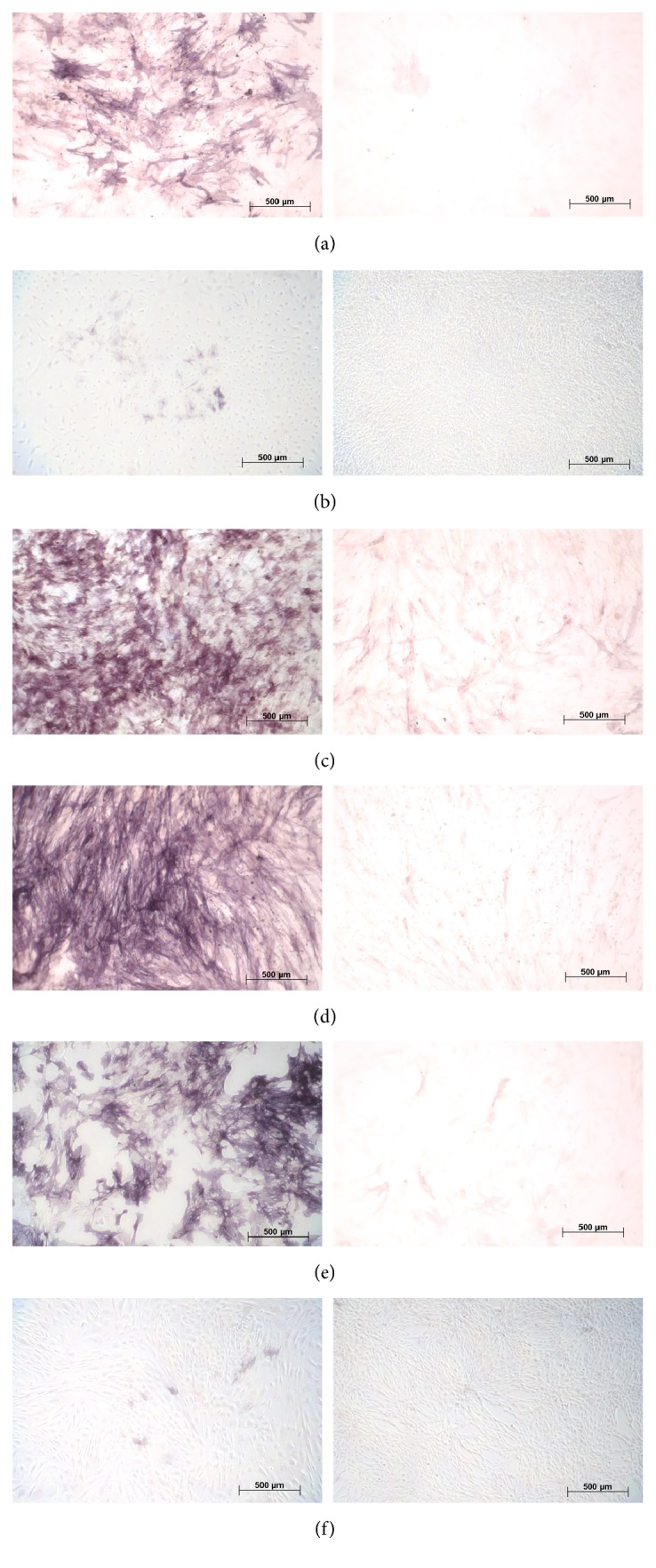
*Osteogenic differentiation of mesenchymal stem cells.* Human mesenchymal stem cells were subjected to osteogenic differentiation for 10 days in culture. (a) Adipose tissue-derived; (b) amniotic tissue-derived; (c) bone marrow-derived; (d) chorionic tissue-derived; (e) liver-derived; (f) umbilical cord-derived. Left panels: positive osteogenic-induced; right panels: negative controls noninduced. Bright field microscopy.

We compared the potential of MSCs from various donor sites to differentiate towards adipogenic, chondrogenic, and osteogenic lineages. We found that MSCs had distinct differentiation potentials depending on the donor site. MSCs derived from adipose tissue (AD), bone marrow (BM), and chorion (CH) demonstrated strong capacities for differentiation towards adipogenic (Figure 4), chondrogenic (Figure 5), and osteogenic (Figure 6) lineages. MSCs from liver (LI) had strong differentiation potential towards chondrogenic and osteogenic lineages, and less but still obvious potential towards adipogenic lineages. MSCs derived from umbilical cord matrix (UC) exhibited very low potential osteogenic differentiation potential, and those MSCs derived from amnion (AM) had very low differentiation potential towards adipogenic and osteogenic lineages, as well as lower chondrogenic differentiation potential compared to MSCs derived from other donor sites.

### 3.4. Cell Viability

**Figure 7:**
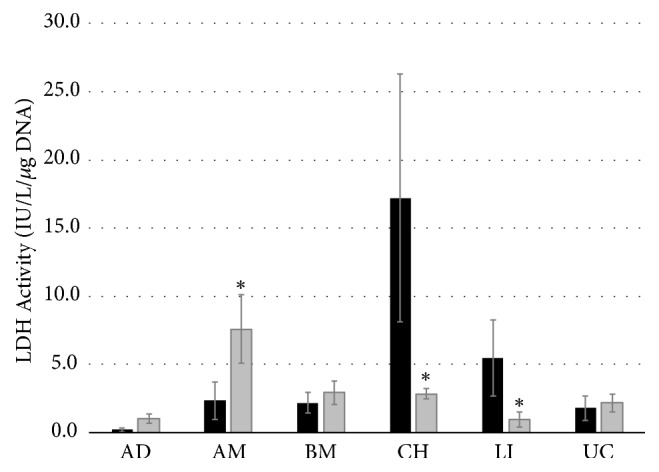
*Lactate dehydrogenase activity.* Lactate dehydrogenase activity as an inverse indicator of cell viability in conventional culture (black bars) and fiber culture (grey bars) of mesenchymal stem cells from adipose (AD) tissue, amniotic (AM) tissue, bone marrow (BM), chorionic (CH) tissue, liver (LI), and umbilical cord (UC). Data are given from 3 biological repeats ± standard deviation. Asterisks indicate statistical significant differences between fiber and conventional cultures with p<0.05.

In order to determine if culture of MSCs in fiber encasement had any potential adverse effects on viability we compared cell disintegration (as LDH activity) in conventional culture with that of fiber culture (Figure 7). We found that AD, BM, and UC MSCs had low LDH activity that was not different between conventional and fiber cultures. CH and LI MSCs had low LDH activity in fiber culture; in conventional culture, LDH activity was significantly higher. AM MSCs had higher LDH activity in fiber culture than in conventional culture.

### 3.5. Analyses of Growth Factor Secretion or Uptake

**Figure 8:**
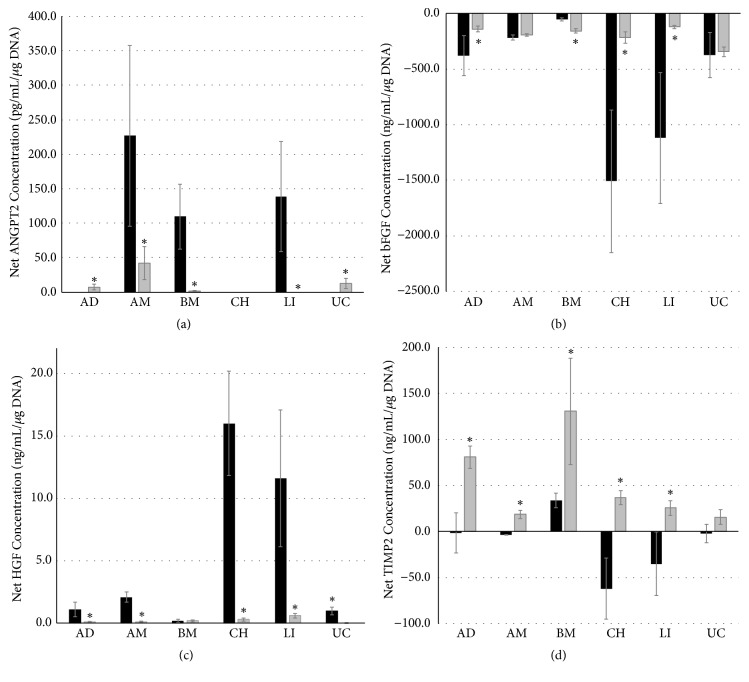
*Enzyme-linked immunosorbent assays.* Net growth factor concentrations in cell culture media of (a) ANGPT2, (b) bFGF, (c) HGF, and (d) TIMP2 of mesenchymal stem cells from adipose (AD) tissue, amniotic (AM) tissue, bone marrow (BM), chorionic (CH) tissue, liver (LI), and umbilical cord (UC) in conventional culture (black bars) and fiber culture (grey bars). Data are given from 3 biological repeats ± standard deviation. Asterisks indicate statistical significant differences between fiber and conventional cultures with p<0.05.

We investigated the secretion or uptake of growth factors from cell culture medium of MSCs from various donor sites in conventional culture and compared these to fiber-encased cultures (Figure 8). In general, MSCs exhibited significant differences in their secretion or uptake of growth factors depending on donor site origin and culture method. We found that blank samples of the complete medium (including antibiotics and antimycotics as well as manufacturer-provided proprietary supplements) contained by average 1.1 pg/mL HGF, 8.5 ng/mL bFGF, and 435.2 pg/mL TIMP2 but no ANGPT2. All data are given as blanked (i.e., net) growth factor concentration per *µ*g DNA.

ANGPT2 secretion (Figure 8(a)) could be measured in conventional cultures of AM, BM, and LI MSCs, but not in cultures of AD, CH, or UC MSCs. The culture in fibers led to an induction of ANGPT2 secretion in AD and UC MSCs, but not in CH MSCs; in AM, BM, and LI MSCs, ANGPT2 secretion was decreased by culture in fibers.

The measurement of bFGF (Figure 8(b)) showed that all cells in all culture conditions took up this growth factor. In all but BM MSC cultures the uptake of bFGF in fiber culture was lower or equal to that in conventional cultures. Conventional cultures of CH and LI MSCs demonstrated the largest uptake of bFGF.

We could detect secretion of HGF (Figure 8(c)) into the medium in all culture conditions by all types of MSCs. Secretion was mostly considerable higher in conventional cultures than in fiber cultures, with the exception of BM MSCs that had equal HGF medium concentrations. The highest concentration of HGF in media of conventional cultures had CH and LI MSCs.

We could measure TIMP2 (Figure 8(d)) secretion in fiber-cultured MSC from all origins, being higher in fiber culture than in conventional cultures. Furthermore, in conventional culture net medium concentrations of TIMP2 were mostly negative, with only BM MSCs showing positive secretion of TIMP2.

### 3.6. Gene Expression Analyses

**Figure 9:**
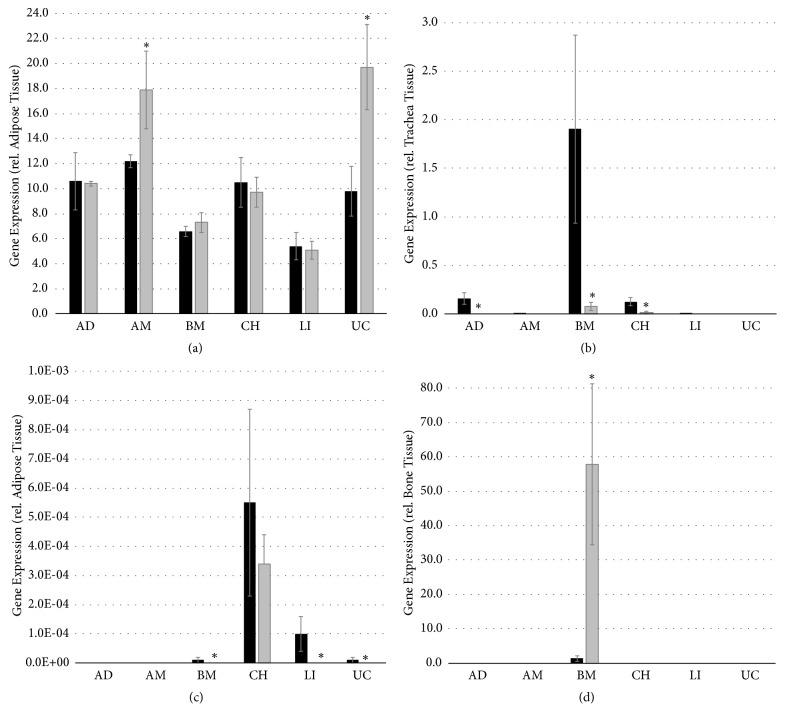
*Gene expression analyses.* Gene expression analyses using real-time PCR for (a) THY1, (b) ACAN, (c) CD34, and (d) DMP1 of mesenchymal stem cells from adipose (AD) tissue, amniotic (AM) tissue, bone marrow (BM), chorionic (CH) tissue, liver (LI), and umbilical cord (UC) in conventional culture (black bars) and fiber culture (grey bars). Data are given from 3 biological repeats ± standard deviation. Asterisks indicate statistical significant differences between fiber and conventional cultures with p<0.05.

In order to investigate potential differences between MSCs from various donor sites as well as possible effects of fiber culture, we analyzed the expression of several MSC-relevant genes (Figure 9). These included genes that are typical mesenchymal-associated (THY1) and those that are lineage-associated (chondrogenic ACAN, adipogenic ADIPOQ, hematopoietic CD34 and PTPRC, as well as osteogenic DMP1).

As expected, THY1 (Figure 9(a)) was strongly expressed by all MSCs with minor differences between MSCs from various donor sites. Compared to conventional cultures, the culture in fibers increased THY1 expression of AM and LI MSCs but did not affect that of other MSCs. The typical adipocyte gene ADIPOQ and mature hematopoietic gene PTPRC could not be detected to be expressed in any of the MSCs. Interestingly, we could observe notable differences in the expression of lineage-specific genes ACAN, CD34 and DMP1. Whereas ACAN expression (Figure (b)) was either undetectable or very low in all MSCs cultured in fibers, conventional cultures of AD, CH and especially BM-derived MSCs had detectable levels. Similarly, low but detectable levels of CD34 expression (Figure 9(c)) could be detected in conventional cultures of BM, CH, LI, and UC MSCs, but only in CH MSCs in fiber culture. DMP1 (Figure 9(d)) could be only detected in BM MSCs; the expression of DMP1 was significantly higher in fiber culture than in conventional culture.

## 4. Discussion

We compared human MSCs from different tissue or organ sites, and the potential effects of fiber encasement on their viability, growth factor secretion, as well as lineage-specific differentiation and gene expression. We found that MSCs exhibited significant differences depending on donor site origin. Effects of fiber encasement varied between MSCs depending on their origin; cell viabilities were either the same or higher when encased, except for amnion-derived MSCs that demonstrated lower viability in encased culture. The encasement of MSCs in semipermeable membranes [26] would offer the advantage of providing an immune barrier [27] but allowing MSCs to release supportive growth factors and cytokines without being permanently integrated in the host environment. After recovery of the patient, encasements could be removed. Differences in study outcomes using immune-compromised animal versus allogeneic human MSCs in a nonimmune-compromised setting were discussed by several authors, suggesting that MSCs could be less immune-privileged than previously thought. Galipeau et al. [28] discussed potential consequences in regard to rejection, and Ankrum et al. [29] pointed towards immune evasive mechanisms. Eliopoulos et al. [30] demonstrated that allogeneic mouse MSCs are immune rejected in immune competent mice. Immunoisolation could therefore be of interest for the use of MSCs for transplantation therapies, including transplantation of genetically engineered allogeneic MSCs for secretion of therapeutic proteins.

Contrary to what one might expect, MSCs isolated from different organs appear to be not equal, and also multiple variables in culture can greatly affect their properties* in vitro* (for reviews, see [10, 31]). Ideally, for a comparison of MSCs derived from different organs, MSCs should derive from the same patient in order to minimize donor variations. Using human samples, however, MSC samples from different organs can rarely be obtained from the same donor, and is virtually impossible when including MSCs of embryonic origin such as umbilical cord, amnion or chorion. As we could observe significant differences between MSCs from different organs derived from different donors, donor variation appeared to be acceptable. Chen et al. [32] demonstrated that proliferation* in vitro *was highest in MSCs isolated from umbilical cord, followed by those from menstrual blood, and was lowest in adipose tissue-derived MSCs. Our data did not show significant difference in population doubling times between all analyzed MSCs. In addition, in their studies gene expression of these MSCs from three different sites exhibited significant differences, which corroborates our findings. A comparison of MSCs derived from adipose tissue and umbilical cord from Choudhery et al. [33] determined similar properties and surface marker expressions; our data also show that MSCs from adipose tissue and umbilical cord have high similarities, but are markedly different to that of MSCs from other sites, such as liver, chorion, or bone marrow. In both studies [32, 33], MSCs from adipose had lowest proliferation potential. Furthermore, the capacity of MSCs to proliferate and differentiate has been shown to be negatively impacted by donor age [34]. For defining MSCs, several surface molecules have been suggested to be present or absent [25]. Positive expression of CD105, CD90 and CD73, and absence of expression of various molecules including hematopoietic CD45 and CD34 are commonly defined for MSCs. Our data mostly corroborate these findings; however, in our studies, MSCs derived from LI and CH demonstrated some CD45 surface expression (5% and 2%, respectively). Although this expression could be indeed an indicator of hematopoietic lineage commitment, a potential contamination with hematopoietic cell types cannot be excluded. CD105 surface expression of MSCs from different sites was significantly diverse and considerably low; we could observe highest percentages in LI MSCs (58%) and lowest in AM MSCs (4%). Although CD105 is mostly considered as a typical MSC marker, there is some inconsistent data about its suitability. For example, the chondrogenic differentiation potential of bone marrow-derived MSCs was shown to be independent of their CD105 surface expression by Cleary et al. [35]. Dizaji et al. [36] found that 76% of amniotic membrane-derived but 92% of adipose tissue-derived MSCs were positive for CD105. Lee et al. [37] demonstrated that CD105 expression of bone marrow-derived MSCs was influenced by culture conditions; alginate as well as transforming growth factor beta3 strongly reduced CD105 expression.

We found that MSCs had distinct differentiation potentials depending on the donor site. Kern et al. [38] observed that MSCs derived from umbilical blood had the lowest adipogenic differentiation potential compared to those isolated from bone marrow or adipose tissue. In our experiments, MSCs derived from umbilical cord matrix exhibited low adipogenic differentiation potential, and those derived from amnion had overall the least differentiation potential. Kim et al. [39] compared umbilical cord-derived with chorion-derived cells, which had mostly similar properties, but umbilical cord-derived MSCs had lower expression of adipogenic genes. Likewise, we could observe less adipogenic differentiation of umbilical cord-derived MSCs compared to chorion-derived MSCs.

For clinical applications, in addition to the ease of donor cell harvest, a further relevant question is the yield of MSCs derived from different donor sites. As found in a comparative review of published studies [40], the percentage of MSCs varies between different donor sites, but also between different donors and even between different harvesting methods. Umbilical cord matrix had highest yields of allogenic MSCs, whereas adipose tissue had the highest yields of autologous MSCs.

Of major interest in clinical transplantation are the supportive properties of MSCs by secreting growth factors and cytokines. Numerous growth factors and cytokines have been found to be secreted by MSCs [8, 9]. We focused on the quantification of four growth factors (angiopoietin-2 (ANGPT2), basic fibroblast growth factor (bFGF), hepatocyte growth factor (HGF), and tissue inhibitor of metalloproteinase 2 (TIMP-2)). These growth factors are known in general to be important for tissue regeneration. We found significant differences in secretion and uptake of growth factors depending on donor sites of MSCs. As shown by Schinköthe et al. [11], from 120 analyzed growth factors and cytokines, TIMP-2, ANGPT2, and bFGF were among the ten secreted at highest concentration of bone marrow-derived MSCs, and HGF also being secreted. We could also observe TIMP2 secretion in conventionally cultured bone marrow-derived MSCs, yet MSCs from all other donor sites took up TIMP2 from culture medium. In contrast, fiber-encased MSCs from all donor sites demonstrated secretion of TIMP2. Contrary to findings from Schinköthe et al., in our experiments bFGF was not secreted by any of the MSCs but taken up from the culture medium. We could detect ANGPT2 secretion in conventional cultures of amnion, bone marrow, and liver-derived MSCs but not in adipose, chorion, and umbilical cord-derived MSCs. HGF was secreted by all MSCs analyzed.

## 5. Conclusions

MSCs derived from different tissues were not equal. Thus, the consideration for selection for a source of MSCs for clinical transplantation should not only depend on availability but also on their distinct properties, such as the capacity to secrete certain growth factors or to differentiate towards certain lineages, which is of relevance for the specific transplantation site. Based on our findings of gene expression and surface marker analyses, undifferentiated bone marrow-derived MSCs already expressed some chondrogenic and osteogenic markers, whereas undifferentiated liver-derived and chorion-derived MSCs showed slight hematopoietic marker expression and therefore might be less suitable than MSCs derived from adipose tissue, amnion, or umbilical cord for transplantation into all sites. The encasement of MSCs into semipermeable membranes could provide a physical immune barrier, allowing for therapy monitoring, preventing cell fusion, and allowing for the removal of the transplanted cells if necessary.

## Data Availability

The data used to support the findings of this study are included within the article.
